# Delivering Prolonged Intensive Care to a Non-human Primate: A High Fidelity Animal Model of Critical Illness

**DOI:** 10.1038/s41598-017-01107-6

**Published:** 2017-04-26

**Authors:** P. Guillaume Poliquin, Mia Biondi, Charlene Ranadheera, Mable Hagan, Alexander Bello, Trina Racine, Mark Allan, Duane Funk, Gregory Hansen, BJ Hancock, Murray Kesselman, Todd Mortimer, Anand Kumar, Shane Jones, Anders Leung, Allen Grolla, Kaylie N. Tran, Kevin Tierney, Xiangguo Qiu, Darwyn Kobasa, James E. Strong

**Affiliations:** 1 Special Pathogens, National Microbiology Laboratory, Public Health Agency of Canada, Winnipeg, Manitoba Canada; 2 Department of Medical Microbiology and Infectious Diseases, College of Medicine, Faculty of Health Sciences, University of Manitoba, Winnipeg, Manitoba Canada; 3 Department of Paediatrics & Child Health, College of Medicine, Faculty of Health Sciences, University of Manitoba, Winnipeg, Manitoba Canada; 4 Department of Anaesthesia and Medicine, College of Medicine, Faculty of Health Sciences, University of Manitoba, Winnipeg, Manitoba Canada; 5 Department of Pediatrics, Royal University Hospital, Saskatoon, Saskatchewan Canada; 6 Child & Women’s Health Program, Health Sciences Centre, Winnipeg, Manitoba Canada; 7 Department of Medicine, College of Medicine, Faculty of Health Sciences, University of Manitoba, Winnipeg, Manitoba Canada

## Abstract

Critical care needs have been rising in recent decades as populations age and comorbidities increase. Sepsis-related admissions to critical care contribute up to 50% of volume and septic shock carries a 35–54% fatality rate. Improvements in sepsis-related care and mortality would have a significant impact of a resource-intensive area of health care delivery. Unfortunately, research has been hampered by the lack of an animal model that replicates the complex care provided to humans in an intensive care unit (ICU). We developed a protocol to provide full ICU type supportive care to Rhesus macaques. This included mechanical ventilation, continuous sedation, fluid and electrolyte management and vasopressor support in response to Ebolavirus-induced septic shock. The animals accurately recapitulated human responses to a full range of ICU interventions (e.g. fluid resuscitation). This model can overcome current animal model limitations by accurately emulating the complexity of ICU care and thereby provide a platform for testing new interventions in critical care and sepsis without placing patients at risk.

## Introduction

Critical care provides an essential, potentially-life saving set of interventions to patients with a wide range of underlying diseases, ranging from exacerbations of chronic disease to acute, life-threatening infections. While there has been a trend toward the reduction of acute care beds since 2000, the opposite has been observed in the number of critical care beds^1^. Further pressure on critical care capacity is expected as the population ages and survival for chronic conditions is prolonged^1, 2^. The leading cause for admission to an intensive care unit (ICU) is infection and sepsis, accounting for approximately 10 to 45% of ICU stays depending on a variety of health system factors^2, 3^. Despite advances in critical care science, septic shock, as recently defined by the Sepsis-3 task force, carries a mortality rate of 35–54%^4^, with sepsis syndromes resulting in 250,000 deaths annually in the United States alone^5^.

The combination of high mortality and heavy resource use has made sepsis an important target of critical care research^6^. Particular attention has been placed on two lines of inquiry. The first has centered on drug development. Several candidate drugs (e.g. recombinant human activated Protein C^7^, tumour necrosis factor-alpha blockade^8^) proved highly promising in animal studies. These positive findings have not had the same impact when tested in humans, as demonstrated by over 100 randomized controlled trials with disappointing results^6, 9^.

A second approach has focused on intensive monitoring and correction of the hemodynamic derangements in sepsis, famously reported in Rivers’ landmark trial of early goal-directed therapy (EGDT)^10^. The study reported survival benefit resulting from early initiation of multi-parameter monitoring followed by rapid therapeutic intervention. EGDT has resulted in controversy with recent multicenter trials finding no significant difference in 90-day mortality, duration of organ support, and length of hospital stay between EGDT-treated patients compared to those who received standard care^11–13^.

The inconsistent benefit provided by the efforts to date speaks to fundamental challenges facing care for critically ill septic patients. One specific challenge is that to date, neither human clinical trials nor animal models have proven to be satisfying research platforms. Human trials face a bevy of challenges, often stemming from the unplanned and rapidly progressive nature of sepsis^14^. There are several well developed small and large animal models of sepsis^9, 15, 16^, each with specific advantages and drawbacks. For example, rodents are readily available and several genetic variants have been developed, however their disease is a poor recapitulation of the human experience, usually compressed on a timescale of hours rather than days to weeks^16^. Dogs have been used as a larger animal model, but provision of intensive care is technically difficult and thus far limited to 96 hours^17^.

The most singular challenge posed by all these models is the difficulty in modelling the complex mix of therapies that are usually bundled when caring for septic patients^17^. Sedative medications that make ventilation possible have negative effects on hemodynamics^17^. Ventilators themselves improve ventilation but can negatively impact venous return to the heart^18^. Such a high fidelity model of critical care would have applications not only to sepsis research but to other aspects of critical care science. We describe a non-human primate (NHP) model of sepsis that, to our knowledge, most closely mimics the conditions and time scale of the human intensive care unit.

## Results

The implementation of this NHP model required overcoming several technical challenges across a range of issues. Each of the four animals yielded individual improvements. The distilled “best practices” are described in the Materials and Methods section at the end of the article. In this section we describe the technical challenges encountered as well as both failed and successful strategies that were employed to resolve them.

### Lessons Learned

#### Line Insertion

Line insertion was accomplished via surgical cut-down in the first animal and percutaneously with ultrasound guidance thereafter. Percutaneous insertion resulted in less pain and healthier wound sites compared to cut-down sites, without impacting function or longevity of the lines. Insertion of lines into the internal jugular veins was attempted on two primates. These were unsuccessful due to skin toughness and jugular vein mobility. As a result, groin sites were preferred.

#### Sedation

Very little information has been published with regard to prolonged sedation in NHPs. One group reported successful sedation of rhesus macaque using 20–50 mg/kg/h of ketamine over a 24-hour period^19^. Based on this information, the pilot experiment employed a combination of ketamine and fentanyl as continuous infusions. The animal was not adequately sedated using this combination, despite high drug doses. As a result, a benzodiazepine was added, initially as intermittent oral diazepam and then as continuous intravenous midazolam. This was partially successful, but boluses of fentanyl, midazolam and ketamine were still frequently needed. This led to the addition of dexmedetomidine as a continuous infusion, resulting in a more relaxed and stable animal. The continuous infusion of ketamine was discontinued for following experiments as it resulted in a dissociative rather than sedated state as well as induced a large amount of saliva that was difficult to clear.

#### Airway Access

Three different methods of airway access were trialed. The pilot animal received a tracheostomy, under the assumption that this would be more stable. Unfortunately, the tracheostomy proved problematic. First, the immature tracheostomy was prone to low grade bleeding and irritation. Second, the distance between the sternal notch and the larynx is substantially longer in the rhesus macaque than in humans. This resulted in an easily displaced tracheostomy tube leading to pneumothoraces and subcutaneous emphysema. Ultimately the primate had to be intubated orally to provide adequate ventilation.

Nasal intubation proved effective in two of the four primates. Nasal intubation is attractive as it reduces oral stimulation and is relatively simple to secure. Once inserted and position was confirmed, the tube could be stitched directly into the nostril and further secured with tape. The biggest drawback is the relative size mismatch between the primate’s nasopharynx and oropharynx. As a result, the tube size that could be passed was relatively small compared to optimal cuff size, resulting in large cuff leaks (~30%). While this did not impact our studies, any studies with significant lung pathology would be difficult to manage with such a large leak.

Finally, oral intubation was employed twice: as a rescue option for one NHP and as the main option for a second NHP whose nasopharynx could not accommodate a large enough nasal intubation. Securing the oral tube was problematic due to the prognathic NHP facial anatomy. A stitch passed through the gum helped improve stability. A further challenge was teeth and jaw strength. One animal bit through the pilot bulb tubing leading to the need for re-intubation. The use of a bite block is therefore recommended.

#### Diurnal Pattern

The first three primates demonstrated a predictable period of arousal twice daily. One would occur near 07:00 daily, while the second occurred in the early evening, ranging from to 18:00–19:00. These periods were heralded by an increase in heart rate, blood pressure and respiratory rate. This increased arousal frequently required sedation boluses. This diurnal pattern has been reported previously^20^. For the fourth animal, we implemented scheduled bolus doses of midazolam (0.1 mg/kg dose) 30–45 minutes prior to the anticipated arousal period. This proved effective in blunting the arousal period. This diurnal pattern faded from day four onward in all animals, an effect also seen in human patients admitted to the ICU^21^.

#### Importance of Checking Lines

There are several lines and extensive tubing involved in this experimental setup. It is important for bedside staff to check the tubing from patient to pump hourly, as tangling and disconnection was a significant risk, particularly with respect to losing the sedation line.

#### Notable Laboratory Differences

The clinicians taking part in this study are trained in human critical care medicine. As a result, they cognitively processed laboratory results using usual human values. This is acceptable for most parameters, as human and macaque reference ranges are similar. A few notable differences are worth highlighting. Rhesus macaques have elevated serum amylase levels with a published range of 333–610 U/L^22^. Using our instruments (Piccolo^TM^), we observed an average of 445 U/L (+/− 225) with no other clinical correlate or ultrasound evidence of pancreatitis. Additionally, Rhesus macaques tend to have a more alkaline blood pH (7.45) due to a lower CO_2_ value (35 mmHg) than humans (pH 7.4 and CO_2_ of 40 mmHg). By contrast, HCO_3_- is similar in both species (range 22–26 mEq/L)^22^.

### Unresolved Issues

#### Feeding & Bowel Care

The organ system with the most unresolved issues is the gastrointestinal system. All four primates developed a paralytic ileus following the initiation of the sedation medications. The gastroparesis was the root cause of two separate aspiration events with resulting pneumonitis or ventilator-associated pneumonia. The constipation led to bloating and abdominal discomfort in at least one animal, leading to an ongoing cycle where increased sedation to compensate for the discomfort worsened the ileus. Several different strategies were tried (including the early initiation of stool softeners, pro-kinetic agents, trophic feeds, continuous feeds, elemental formula, and suppositories) but to date we have been unable to resolve this issue. A trial of enteral methylnaltrexone is planned for future experiments.

#### Sedation Medication Withdrawal

Any experimental design where the animal would be awoken at the end of the trial period would need to consider withdrawal management from the narcotics employed. This would need to include a long-acting oral medication due to the protracted tapering course.

## Discussion

Both large and small animal models of sepsis have been previously published^9, 15, 16^. While each has clear advantages and drawbacks, some capability gaps are present across the various model systems. Some of these gaps include feasibility of serial sampling of body fluids, measuring accurate vital signs and ease of administering multiple simultaneous treatments.

With regard to body fluid sampling, the most common approach involves serial sedation of the NHPs at pre-determined times^23, 24^. The main advantage of this approach is the ability to maintain normal physiology. Important drawbacks include sedative medication side effects, the stress of anaesthesia and lack of flexibility in sampling times^25^. An alternate approach involves training NHPs, for example using the Positive Reinforcement Technique, to present themselves for sampling. This approach can minimize the physiological effects of sampling through fear reduction while removing the need for sedation. Unfortunately trained NHPs require extensive pre-experiment investment and demonstrate variable compliance^26^.

Vital signs are often measured at the time of sedation, introducing distortions due to anaesthetic effects. An alternate approach is implantable telemetry. There exists a range of devices with variable capabilities^20, 27^. They appear to provide accurate and continuous data, but are costly and require pre-experiment implantation followed by a substantial recovery period. A final challenge centres on timely administration of medications. Implanted catheters in trained animals have been used successfully, but are limited by the number of lumens and by animal compliance^20^. Treatment approaches that would involve more than one drug become much less feasible.

The most closely related approach to our model involved a combination of invasive telemetry with an implanted central venous catheter and a trained NHP infected with Zaire ebolavirus^20^. This allowed continuous vital sign monitoring, daily sampling and fluid bolus administration via the catheter. Unfortunately, timely administration of fluids proved to be a logistical challenge, with investigators describing significant delays between recognizing the need for fluid and the delivery time. Full ICU-type care would thus be difficult using this approach.

Our model provided solutions to the above challenges by using human ICU equipment and approaches. Continuous sedation enabled long-term monitoring and avoided the compressed sepsis time-scale typical of small animal models^16^. Multi-site vascular access allowed for serial sampling at any experimental time point. The extensive vascular access allowed for the provision of several different treatments at one time (e.g. fluid, vasopressors and antibiotics). Long term respiratory support through mechanical ventilation was also shown to be possible. This multi-modal approach to treatment thus mirrors the complex care environment seen in human ICUs.

The high-fidelity nature of this model also results in some important limitations. First, the hemodynamic effects of long term intubation and sedation are significant. Mechanical ventilation can potentiate adverse heart–lung interactions necessitating earlier vasoactive medications than in a non-ventilated animal^18^, while most sedation drugs lower blood pressure. A second major challenge is the large capital investment and operating costs necessary to use this model. Recognizing that individual site costs would vary based on a variety of factors, our initial equipment costs were approximately 135,000 USD and an additional 100,000 USD for imaging devices (xray, ultrasound). The operating cost for consumables was 10,000 USD per experiment excluding the cost of the animals. Staffing costs are an additional consideration, though will vary widely depending on staff type and local institutional regulations. When compared to investment in large, multicentre trials that fail to meet expectations, however, this approach may prove cost effective. Finally, the ethical use of NHPs in science is complex. This model maximizes comfort and minimizes pain through the use of continuous sedation and analgesia. Furthermore, the amount of data that can be captured and the flexibility of experimental design can likely reduce overall animal numbers needed for experiments.

It is worth noting that the adverse effects of mechanical ventilation and sedation are an essential challenge of both sepsis management and critical care in general. The use of our model represents an opportunity to test any new intervention under the full complexity of critical care. Any potential intervention or drug that demonstrated efficacy under such conditions would stand a high chance of demonstrating benefit in human trials as well.

## Materials and Methods

The model described herein was refined over the course of four separate experiments. The initial animal was not infected, whereas the other three were infected with our pathogen virus of interest (Makona Ebolavirus, GenBank KT013256.4). The model described below represents the distillation of the approaches that resulted in best performance.

### Pre-experiment Preparation

The Rhesus macaque (*Macaca mulatta*) was chosen for two reasons. It is a useful animal surrogate to human Ebola virus disease^28^ and animals of at least ten kilograms are reasonably common. This size is important to allow the use of commercial paediatric life support equipment. NHP use was authorized under Animal Use Document number H-14–003 following review by the National Microbiology Laboratory Animal Care Committee. This committee operates under the oversight of the Canadian Council of Animal Care, which sets standards and guidelines for animal care in research settings in Canada.

The primates were housed at the National Microbiology Laboratory, Winnipeg, Canada for up to three months ahead of the experiment. A physical exam was performed upon arrival to ensure the animals were healthy. A 10–15 cc/kg blood draw was performed at least two weeks ahead of the experiment. This was used for baseline blood work while the remainder was stored as a potential autologous blood transfusion.

Continuous bedside care was required throughout the experiment. Staffing was provided by four teams rotating through three eight-hour shifts. Each team included at least one veterinary technician, with the remaining members made up of Biosafety Level-4 (BSL4) trained scientists. For safety and care delivery reasons, two bedside caregivers were present at all times; one caregiver attending the animal, while the second was available to perform tasks (e.g. administer medications, run diagnostics). One physician with experience in critical care was in-house at all times, rotating on 12-hour shifts. Additional support was available from respiratory therapists, nurses and pharmacists either in-house or by phone. Furthermore, specialized services such as echocardiography, ultrasonography and radiology were available on an on-call basis.

Our experimental requirements were unique due to the necessity of BSL4. As a result, we had four individuals per team rather than two to ensure sufficient time out of BSL4. It was determined that it would be advantageous to train existing BSL4 staff in basic ICU nursing skills, rather than train ICU nurses in high containment due to the time needed to train and maintain BSL4 skills. A three month nursing skill training programme was offered to the study staff ahead of the experiments.

### Initiation of the Experiment

#### Preparation of the Room and Safety

Animal studies were performed under BSL4 conditions and approved by the Canadian Science Centre for Human and Animal Health Animal Care Committee following the guidelines of the Canadian Council on Animal Care. The room layout mirrored a typical ICU with most equipment concentrated near the head of the animal. The equipment list can be found in Table 1. The room was decontaminated for initial setup. During this phase, bedside care workers handling the NHPs donned appropriate personal protective equipment (PPE), as well as showered and changed prior to exiting the animal space. This was necessary to reduce risk of zoonotic infection (e.g. Herpes B)^29^. Specific to our experiments, PPE needs escalated to BSL4 procedures to perform the infection and care thereafter.

**Table 1:** Clinical Care and Diagnostic Equipment List.

Device Name	Device Purpose
**Clinical Care Equipment**	
Intellivue MX800 Patient Monitor	Flexible monitor platform for continuous monitoring of vital signs
Avea Ventilator	Provision of mechanical ventilation. Able to deliver multiple ventilation modes
Alaris SE Volumetric Pump	Pump designed for delivery of larger volumes of medicine and/or delivery of fluid
Alaris CC Syringe Pump	Pump designed for delivery of small volume infusions; used primarily for continuous sedation medicine delivery
HotDog Veterinary Patient Warming System	Heating system used to help maintain the animal in a stable temperature range
**Diagnostic Equipment**	
ABL 80 Flex Blood Gas Analyzer	Provides analysis of both arterial and venous blood gas
Piccolo Blood Chemistry Analyzer	Point-of-care device for analysis of blood counts and blood chemistry Cartridges employed: Metlac 12 Biochemistry Plus
Vetscan HM5	Point-of-care device aimed at the veterinary market; used for complete blood counts
Aviva Accu chek	Point-of-care device for measurement of blood glucose
Soyee Portable High Frequency SY-HF 102 Xray Source	Used for plain film Xray acquisition
Konica Minolta ImagePilot M24 System	Digital xray development station; also used for visualizing xrays
EDAN SE-1200 EKG Machine	Used for acquisition of electrocardiograms
Philips CX50 Ultrasound	Ultrasound machine used for formal ultrasound and echocardiography examinations
Sonosite M-Turbo Ultrasound	Ultrasound machine used for informal bedside scans (e.g. scanning bladder to look for urinary retention)

Listing of clinical and diagnostic equipment used during this study.

#### Surgical Care of the Animal

The NHPs were initially sedated with intramuscular ketamine/xylazine in the animal care area then transferred to the pre-surgical holding area. The animals’ abdomen, groin and mouth areas were shaved, after which the animal was moved into the surgical area. There, additional sedation was started using isoflurane via face mask (5% for induction and 2–3% for maintenance using oxygen as the carrier gas). From this point on it was necessary to use a warming blanket as sedation appeared to blunt the NHPs’ thermogenesis. Surgeons and/or intensive care specialists proceeded with percutaneous groin insertion of both arterial and central venous catheters under ultrasound guidance. A redundant arterial and central venous line was also inserted in the opposite groin due to the inability to safely insert new lines under BSL4 conditions. Work with other infectious agents may not require these second lines up front.

Once line access was established, continuous sedation was initiated. Following start of intravenous sedation, the airway was secured, preferably through nasal intubation. When nasal intubation was not possible due to the size of the nasal bone orifice, oral intubation was used as an alternative.

### Day-to-day Care

#### Attendance at the Bedside

As part of continuous bedside care, staff were tasked with general as well as specific hourly checks to monitor the primate’s clinical status, equipment function (e.g. line site evaluation) and drug levels (see Table 2). In addition to hourly checks, staff were responsible for husbandry care (e.g. position changes, suctioning, mouth, eye, genitourinary and bowel care).

**Table 2:** Bedside Team Task Checklist.

Hourly Tasks	Task Sign Off		Miscellaneous Notes
Vitals	01:00 □	13:00 □	
Check restraints	02:00 □	14:00 □	
NG location	03:00 □	15:00 □	
Cuff pressure for ventilation at 9cc	04:00 □	16:00 □	
	05:00 □	17:00 □	
Check line sites	06:00 □	18:00 □	
Check urinary catheter site	07:00 □	19:00 □	
Ensure pressure bags at 300 mm Hg	08:00 □	20:00 □	
Check HotDog (on and set temp)	09:00 □	21:00 □	
Record vent settings	10:00 □	22:00 □	
Record all Ins	11:00 □	23:00 □	
Record all Outs	12:00 □	24:00 □	
**Q 3 Hourly Tasks**	**Task Sign Off (record time done)**		**Miscellaneous Notes**
NG pH and Volume Turn NHP, and shift up bed (After recording vitals)	____ □	____ □	
	____ □	____ □	
	____ □	____ □	
	____ □	____ □	
**Q 4 Hourly Tasks**	**Task Sign Off (record time done)**		**Miscellaneous Notes**
Rinse feed bag and add feed	____ □	____ □	
	____ □	____ □	
	____ □	____ □	
Eye and mouth care	____ □	____ □	
	____ □	____ □	
	____ □	____ □	
	____ □	____ □	
**Q 8 Hourly Tasks**	**Task Sign Off (record time done)**		**Miscellaneous Notes**
Capillary blood glucose Using dipstick to check urine sample	____ □		
	____ □		
	____ □		
**Q 12 Hourly Tasks**	**Task Sign Off (record time done)**		**Miscellaneous Notes**
Re-zero CVP and Art lines Clear pumps (including feeding pump)	00:00 □	12:00 □	
**Once Daily Tasks**	**Task Sign Off (record time done)**		**Miscellaneous Notes**
Change feed bag	____ □		
Record Abdominal Girth	____ □		
Chest x-ray	____ □		
Abdominal x-ray	____ □		
EKG	____ □		
MetLac 12 (Picollo)	____ □		
Biochem Plus (Picollo)	____ □		
Art Gas (ABL80)	____ □		
Venous gas (ABL80)	____ □		
CBC (HM5)	____ □		
VetScan	____ □		
Swabs (Ocular, Nasal, Oral, Sweat, Rectal)	____ □		
Urine sample for virology	____ □		

#### Vital Signs and Laboratory Monitoring

Vital signs were monitored continuously using sensors tied into a cardiorespiratory monitor. Heart and respiratory rate were monitored via skin electrodes. Arterial blood pressure was monitored via a transduced arterial line, with a conventional non-invasive blood pressure cuff available as backup and confirmation. Central venous pressure was monitored via a transduced central venous line port. End-tidal CO_2_ was measured via a sensor added in-line to the ventilator circuit. Oxygen saturation was measured using clip-like probe pulse oximeter. Temperature was monitored by rectal probe. Built-in sensors in the ventilator provided continuous monitoring of respiratory rate and pressure/volume delivery. Intermittent use of 12-lead electrocardiographs and echocardiograms were used to provide more in-depth analysis of cardiac function. Table 3 presents the range of vital signs we observed under our experimental conditions prior to infection. Laboratory monitoring was performed regularly using point-of-care diagnostic machines (Table 1). The machines performed well using NHP blood despite being designed for human samples.

**Table 3:** Range of Vitals Observed Pre-infection.

	Average Value	Range
**Systolic Blood Pressure (mmHg)**	125 (SD 12.5)	96–156
**Diastolic Blood Pressure (mmHg)**	71 (SD 6.1)	39–109
**Mean Arterial Pressure (mmHg)**	92 (SD 8)	59–144
**Heart Rate (beats/minute)**	109 (SD 9.6)	55–170
**Respiratory Rate (breaths/minute)**	21 (SD 1)	14–33
**Oxygen saturation (%)**	97 (SD 1)	91–100
**End Tidal CO**_**2**_	39 (SD 2.1)	31–52
**Temperature (including surgical time) (°C)**	36.0 (SD 1)	33.3–39.3
**Temperature (excluding surgical time) (°C)**	38.0 (SD 1)	37.1–39.3

Overview of observed vital sign ranges. It is important to consider that all blood pressure and heart rate values are potentially affected by sedation medications. Additionally, the lower range of the respiratory rate was set by the ventilator since a minimum rate was set on the machine. Similarly, supplemental oxygen and changes to ventilator settings would have affected O_2_ saturation and end tidal CO_2_ values. A final consideration is body temperature, which was affected by artificially low ambient temp.

#### Ventilation

Ventilation was delivered using a pressure-regulated volume controlled strategy. A ventilator-associated pneumonia prevention approach was used (i.e. 15° elevation of the head of the bed, body positioning turns every two to four hours, etc.). Continuous oxygen saturation and end-tidal carbon dioxide monitoring was used for gauging adequacy of ventilation in real-time. Blood gas measurement using both arterial and venous samples was used periodically as well. Our ventilator setting ranges under steady state were: PEEP 5–8, PIP 16–20 (tolerated up to 28), tidal volume 8–9 ml/kg, rate 17–20 (tolerated up to 30). Endotracheal cuff leak ranged from 10–40%; values above 40% indicated either tube dysfunction or debris in the tube. Scheduled and as-needed use of in-line suction to clear the endotracheal tube of secretions was performed.

#### Fluids, Feeding, Bowel & Bladder Care

Initial fluid administration rate was measured using the pediatric estimate of 4 cc/kg/h for the first ten kilograms and 2 cc/kg/h for additional mass. As disease progressed, renal and gastrointestinal dysfunction became prominent. When this occurred, fluid management was changed to replacing calculated insensible losses (300–400 ml/m^2^/day) plus measured losses in four-hour blocks. We primarily used D5W 0.9% saline solution, occasionally replaced with D5W 0.45% saline or Lactated Ringers based on biochemistry results. Potassium chloride supplementation was given based on blood potassium value and renal function. All intravenous access ports were flushed periodically to prevent clot formation and locked with heparin containing saline (100 units/ml). Urine output was measured on an hourly basis through the use of a Foley catheter and a graduated urine bag. While it proved ineffective due to opioid-induced gastroparesis, we planned for and attempted feeding the NHPs through nasogastric means. Bowel movements were infrequent due to the effect of the sedation drugs. Stool softeners were tried with uncertain efficacy (see Table 4).

**Table 4:** Drug List and Dosing Range.

Drug Name	Route of Administration	Dosing Range
**Sedation Medications**		
Fentanyl Continuous Infusion	IV	7–11 µg/kg/h
Fentanyl Bolus Dose	IV	1–3 µg/kg/dose
Midazolam	IV	0.3–0.8 mg/kg/h
Midazolam Bolus Dose	IV	0.1–0.3 mg/kg/dose
Dexemedetomidine	IV	0.4–1 µg/kg/h
Ketamine Infusion*	IV	20–30 µg/kg/min
Ketamine Bolus Dose	IV	1–4 mg/kg/dose
**Vasoactive Medications**		
Epinephrine	IV	0.05–0.7 µg/kg/min
Norepinephrine	IV	0.4–0.7 µg/kg/min
Vasopressin	IV	1–1.15 mUnits/kg/min
**Other Medications**		
Piperacillin/Tazobactam	IV	300 mg/kg/dose of the piperacillin component every 6 hours
Meropenem	IV	10–20 mg/kg/dose every 8 hours
Hydrocortisone	IV	1–2 mg/kg/dose every 6 hours
Metoclopromide	IV	0.1 mg/kg/dose
Lactulose (10 g/15 ml formulation)	NG/NJ	20 ml every 12 hours
Senna 8.8 mg/5 ml formulation	NG/NJ	5 ml every 12 hours

*Long-term ketamine infusion is not recommended due to a tendency to induce an unstable dissociative effect rather than sedation.

### Pharmacology

Due to a paucity of information regarding the pharmacodynamics of many continuous infusion drugs in NHPs, the dosing ranges were derived primarily from a titration-to-effect approach (Table 4).

#### Sedative Medications

The sedation goal was defined as a sedated animal with preserved cough response and an appropriate response to handling (determined by a transient increase in HR and BP). This goal was best achieved using continuous infusions of fentanyl, midazolam and dexmedetomidine. All three animals followed a similar pattern whereby infusion doses were relatively stable for the first 12 hours (with associated relative bradycardia and hypotension), followed by rapid escalation over the next 12–24 hours until a steady state was achieved. Dose escalation related to tachyphylaxis occurred about once per 24 hour thereafter. Even with a steadily sedated animal, periods of transient increase in arousal were common. These resulted either from external stimulation (e.g. position changes, echocardiography, loud noises) or internal noxious stimuli (e.g. coughing, deep suction, circadian rhythms). Regardless of cause, both responded well to bolus doses of fentanyl and/or midazolam. Fentanyl boluses were best for noxious stimuli such as pain, while midazolam was more effective for stimulation from increased handling.

As a safety measure, ketamine doses were on standby to sedate the animal if other boluses failed. The ketamine was available in two different forms: a syringe pump connected to a central line and in pre-measured syringes for “push” delivery through a line hub. Sedation was aided through environmental interventions (e.g. eye covers, earplugs, quiet environment). Four-point soft restraints were applied to the animal to prevent accidental disconnection of equipment during transient arousals (e.g. while coughing, retching).

#### Other Medications

There were fewer opportunities to use non-sedation drugs, resulting in limited information. The antibiotics piperacillin-tazobactam and meropenem were used for presumptive ventilator-associated pneumonias. Epinephrine, norepinephrine and vasopressin were used with a titration-to-effect approach using BP and HR responses to determine efficacy. The maximum doses were defined based on the lack of a further HR or BP response. This may have been due to the animal being in a state of refractory septic shock. Hydrocortisone (1–2 mg/kg/dose every 6 hours) was also provided for fluid and catecholamine-resistant shock.
